# Growth rate-coordinated transcriptome reorganization in bacteria

**DOI:** 10.1186/1471-2164-14-808

**Published:** 2013-11-20

**Authors:** Yuki Matsumoto, Yoshie Murakami, Saburo Tsuru, Bei-Wen Ying, Tetsuya Yomo

**Affiliations:** 1 Graduate School of Information Science and Technology, Osaka University, 1-5 Yamadaoka, Suita, Osaka 565-0871, Japan; 2 Graduate School of Life and Environmental Sciences, University of Tsukuba, Tsukuba, Ibaraki 305-8572, Japan; 3 Graduate School of Frontier Biosciences, Osaka University, Suita, 1-1 Yamadaoka, Suita, Osaka 565-0871, Japan; 4 Exploratory Research for Advanced Technology (ERATO), Japan Science and Technology Agency (JST), Suita, Osaka 565-0871, Japan

## Abstract

**Background:**

Cell growth rate reflects an organism’s physiological state and largely relies on the ability of gene expression to respond to the environment. The relationship between cellular growth rate and gene expression remains unknown.

**Results:**

Growth rate-coordinated changes in gene expression were discovered by analyzing exponentially growing *Escherichia coli* cells cultured under multiple defined environments, in which osmotic pressure, temperature and starvation status were varied. Gene expression analyses showed that all 3,740 genes in the genome could be simply divided into three clusters (C1, C2 and C3), which were accompanied by a generic trend in the growth rate that was coordinated with transcriptional changes. The direction of transcriptional change in C1 indicated environmental specificity, whereas those in C2 and C3 were correlated negatively and positively with growth rates, respectively. The three clusters exhibited differentiated gene functions and gene regulation task division.

**Conclusions:**

We identified three gene clusters, exhibiting differential gene functions and distinct directions in their correlations with growth rates. Reverses in the direction of the growth rate correlated transcriptional changes and the distinguished duties of the three clusters indicated how transcriptome homeostasis is maintained to balance the total expression cost for sustaining life in new habitats.

## Background

The growth rate of cells represents their physiological status, and cellular physiology largely relies on gene expression. Consequently, gene expression is believed to be related to growth rate [1]. In bacterial cells, growth rate-associated gene expression is known to be related to ribosome biosynthesis [2,3] and the level of RNA polymerase [4,5], and growth rate-related gene expression has been reported in relation to carbon, nitrogen and sulfur utilization [6-8]. In yeast cells, growth rate-coordinated gene expression has been reported to be affected by the interplay between the stress signal of SAPK (stress-activated protein kinase) and the growth signal of TOR (target of rapamycin) [9], which both antagonistically regulate the expression of growth- and stress-related genes [10]. In addition, yeast cell transcriptome analyses identified genes that are correlated with growth rate, with both positive and negative associations [11-14]. Despite intensive study, the conclusions reached thus far have been limited to describing a number of genes with particular functions, and the studies have been restricted to examining experimental conditions under which cells were grown with depleted resources.

Nevertheless, a correlation between growth rate and gene expression has been assumed universal across the genome regardless of environmental variations. That is, the patterns of global transcriptional changes could be independent of the types of environmental stresses. This assumption was partially supported not only by the observation of negative epistasis in bacterial transcriptome reorganization in response to environmental and genetic perturbations [15], but also the finding of hundreds of overlapped genes with core stress responses in yeast [16-18]. However, global transcriptional changes have been investigated to establish the rules of stress responses (*i.e.*, pulse-like transcriptome reorganization), and the results have provided fruitful insights into the specific patterns of gene expression that occur in response to particular external perturbations [19-24], but without any linkage to the growth rate. Thus, it remains necessary to determine the general relationships between growth rate and global gene expression (*i.e.*, by screening the transcriptome).

To correlate the transcriptome with the growth rate, it is crucial to analyze the gene expression in cells that are growing exponentially at a constant growth rate rather than in responsive cells during stress response. Intermediate changes in gene expression in response to stress were accompanied by decreased growth, and this response might differ from the changes in gene expression that correspond to constant growth at a reduced rate (Additional file 1: Figure S1). Transcriptional changes in response to pulse-like perturbations, such as heat shock, osmotic shock, and peroxide, did not show any significant correlation to growth rate [25]. The dissimilarity among these studies is believed to be caused by divergent time scales; in particular, differences in the time exposure to changed environments (*i.e.*, stress), the speed of gene regulation in response to stressors, and the lag time between changes in transcription and growth (Additional file 1: Figure S1). Differences in environmental controls and/or experimental design (*e.g.*, in timing measurements) could lead to contradictory results.

To investigate the potential relationship between gene expression and growth rate, exponentially growing *Escherichia coli* cells were cultured in multiple types of defined environments and examined. The regulatory mechanisms corresponding to gene expression and stress conditions are generally highly related, such as the heat shock activated *rpoH* regulation [26], the general stress response induced *rpoS*[27,28], and the stringent response mediated by ppGpp [29,30]. Because these mechanisms generally involved different genes and varied regulatory network sizes, the following environmental controls were varied osmotic pressure, temperature and starvation. These environmental conditions contribute to cell growth and proliferation, and accordingly affect global gene expression greatly. Investigating the gene expression pattern responsible for constant growth rate rather than changes in response to stress would further promote strategies for achieving cell sustainability under changing environments. In brief, our analyses successfully linked the global architectures involved in transcriptional changes and exponential growth rate (*i.e.*, the magnitude of physiological activity), and presented an evident correlation between changes in gene expression and positive or negative growth rates. The results emphasize not only the reorganized gene expression patterns that are responsible for sustainable growth under varied environments but also offer valuable hints to understanding how the cell transcriptome is adjusted in a universal manner when organisms expand their ecological niche.

## Results

### Constant exponential growth under diverse environments

**Figure 1:**
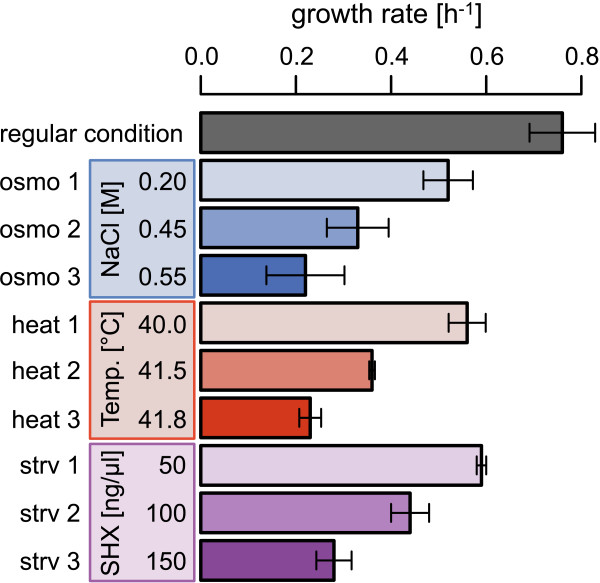
**Growth rates under various conditions.** Grey, blue, red, and violet represent growth under regular, osmotic pressure (osmo), high temperature (heat) and starvation (strv) environments, respectively. Differences in color saturation represent different growth rates.

To experimentally link constant growth and gene expression patterns, the growth rates of exponentially growing cells were determined under varied conditions. Because the present study focused on the exponential growth phase (*i.e.*, gene expression at a constant growth rate), the unpredictable delay and/or time lags related to changes in both gene expression and growth could be neglected (Additional file 1: Figure S1). Cell growth took place under defined conditions. Three types of environmental controls were applied, that is, osmotic variety (osmo 1–3), temperature diversity (heat 1–3) and starvation (strv 1–3). Cell growth within the exponential phase under precisely controlled culture conditions was repeatedly tested to find quantitative relationships between growth rates and external conditions (Additional file 1: Figure S2). Finally, ten conditions comprising three culture conditions for each environmental control and the standard condition (Figure 1) were analyzed. The cells experienced comparable growth rates under even the most diverse culture conditions, for instance, cell growth in the presence of 0.55 M NaCl (osmo 3) was approximately equivalent to growth at 41.8°C (heat 3). These results clearly reconfirmed that the cells could attain equivalent growth rates in different types of environments. The precisely evaluated growth rates quantitatively reflected both the physiological activity of the exponentially growing cells and the magnitude of the continued stress from these environments.

Gene expression was evaluated using a high density tiling array as previously described [15], and three replicates from each independent culture were performed for each growth condition. Mean values of the three replicates were used for the transcriptome analyses that followed (Additional file 1: Figure S3). The final expression data sets corresponded to 3,740 genes and were analyzed by removing repeated gene names from all 3,778 genes that were assigned to the *E. coli* strain used in the present study.

### Similarities in transcriptional changes regardless of environmental variation

Gene set enrichment analysis (GSEA) [31] was performed to evaluate the significance of transcriptional changes at the gene group level. Two categories of gene groups were employed: the gene category [32], which was clustered according to gene function and the transcriptional network (*i.e.*, transcriptional factors, TFs) category [33], which is based on modes of gene regulation.

**Figure 2:**
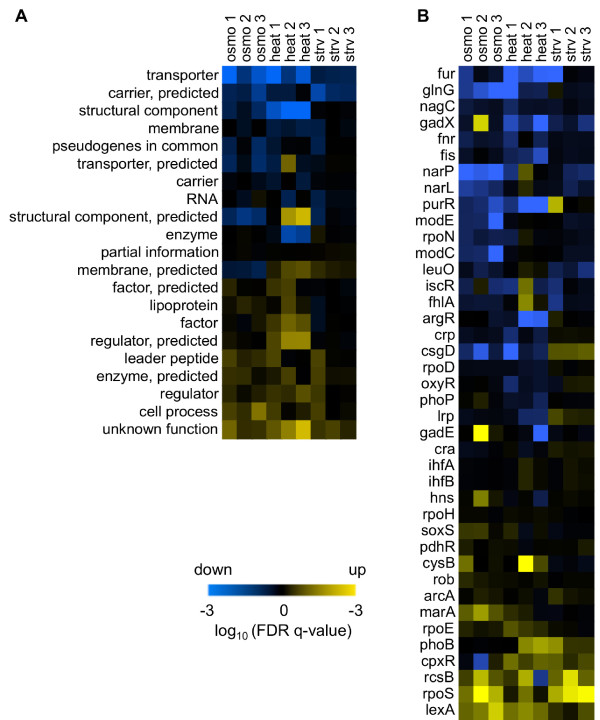
**Similarities in transcriptional changes.** The GSEA results are shown as heat maps. Two types of annotations were used to enrich the gene categories **(A)** and the transcriptional networks (TFs, **B**). The statistical significance (FDR *q*-value) of the transcriptional changes in the TF and gene categories is represented by the gradation from dark brown to yellow or blue on a logarithmic scale. Vivid colors represent high significance in the directions of either upregulated (yellow) or downregulated (blue) genes. The environments responsible for the transcriptome are indicated as follows: regular, osmotic pressure (osmo), temperature (heat), and starvation (strv). The numbers following the environmental abbreviations represent differences in growth rates, as indicated in Figure 1 with higher numbers equivalent to slower growth.

Gene categories showing significant transcriptional changes were identified by comparing gene expression under regular conditions with expression under the other nine conditions. The statistical significance of transcriptional changes at the gene category level is represented using a heat map (Figure 2A). Similar gene expression changes were identified independent of environmental diversity. For instance, genes in the unknown function, cell process and regulator categories exhibited upregulated expression, whereas genes in the transporter and structural component categories exhibited downregulated expression (Figure 2A). A consistent analytical result was acquired with respect to the TFs, and this result was based on the same comparison. For example, the transcriptional networks regulated by *lexA* and *rpoS* appeared to be induced whereas those controlled by *fur* and *glnG* appeared to be suppressed, regardless of the external conditions (Figure 2B). The two relevant heat maps provide a global view of transcriptional changes taking place in both gene category and gene regulation and capture the overlapped transcriptional changes in common, regardless of the environmental specificity. This finding was supported by data sets relating to the stress response of *E. coli* cells under an assortment of environmental perturbations [24] (Additional file 1: Figure S4).

### Genes categorized according to correlated transcriptional change dynamics

**Figure 3:**
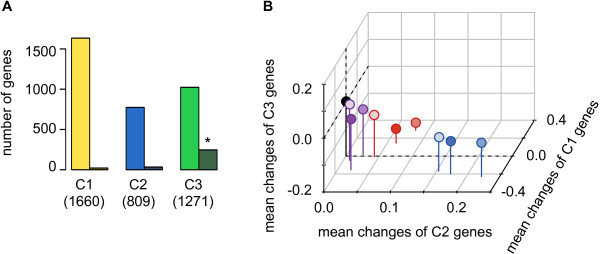
**Genes having similar transcriptional change dynamics. A**. Gene clusters. The three gene clusters determined by *K*-means clustering are shown in yellow (C1), blue (C2), and green (C3). The number of genes in each cluster is indicated. The essential genes comprising the three clusters are shown in dark green. An asterisk indicates the statistical significance (*p* < 0.01) of the biased number of essential genes within cluster C3, as evaluated using binomial tests. **B**. The three-dimensional space formed by the three clusters. Clusters C1, C2, and C3 are represented by the axes *z*, *x*, and *y*, respectively. The lines representing zero on the three axes are indicated using dashed lines. Gene expression under regular conditions is marked in black and located at the zero points of all three axes. Gene expression under the three studied environmental types having varied growth rates are indicated separately. The use of color hue and saturation are as described in Figure 1: osmotic pressure (blue), temperature (red), and starvation (violet).

A *K*-means clustering analysis [34] was subsequently performed to categorize genes with similar dynamics in transcriptional changes among all conditions. All 3,740 genes were manually divided into three clusters (*i.e.*, *K* = 3), as other cases of varied *K* values, *i.e.*, *K* = 16, provided the equivalent conclusion (Additional file 1: Figure S6). Genes classified within the same cluster exhibited similar patterns (coherent dynamics) in transcriptional changes under the nine culturing conditions. In sum, 1,660, 809, and 1,207 genes were clustered in C1, C2, and C3, respectively (Figure 3A), without considering the environmental specificity or the growth rate. Cluster C3 included significant numbers of essential genes (*p* < 0.01).

A three-dimensional space could be formed by considering groups C2, C3, and C1 as the *x*, *y,* and *z* axes, respectively (Figure 3B). Transcriptional changes in genes from the same cluster under individual culture conditions were averaged to generate a mean value as the representative transcriptional change of the corresponding gene cluster under the defined conditions. Ten positions (*i.e.*, culturing conditions) with three representative values of C1, C2, and C3 were acquired. The representative transcriptional changes for C1, C2, and C3 under the regular condition were all zero (Figure 3B, black) because they provided the base values for the transcriptional change calculations. The positions of these nine culture conditions appeared to be occupied within a limited space; that is, the range of decrease C3 (< 0) and increased C2 (> 0) across all nine environments (Figure 3B). Furthermore, these condensed localizations on the C2 and C3 axes somehow exhibited reversed directions to one another; that is, increase on the C2 axis was always correlated with decrease on the C3 axis. This reverse relationship between C2 and C3 must have triggered restrictive localization in the three-dimensional transcriptional change space.

### Differentiation in gene function and regulation among the three clusters

Significantly (*p* < 0.01) enriched gene functions and regulations in the three clusters were examined. The biological functions of the genes that were significantly concentrated in clusters C1, C2 and C3 were identified according to their annotations in GO terms [35,36], gene categories [32], and MultiFun [37], respectively.

**Figure 4:**
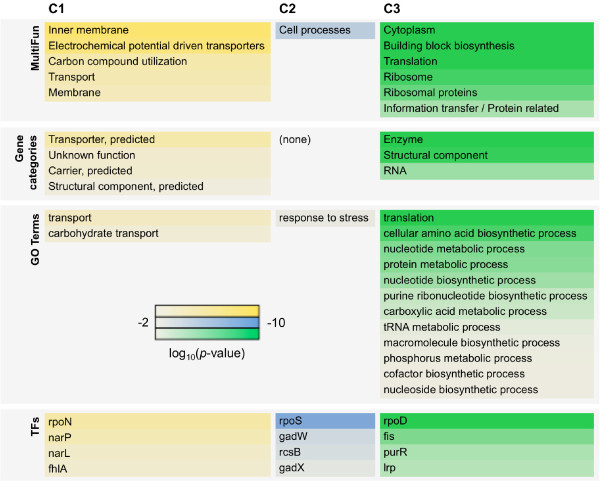
**Differentiated gene functions and regulations.** Gene functions are designated using MultiFun, gene categories, and GO terms. The gene regulations are based on transcriptional networks (TFs). All annotations were made according to public data banks as described in the main text. C1 (yellow), C2 (blue) and C3 (green) represent the three gene clusters determined in Figure 3, and color bars indicate the statistical significance in log-scaled *p* values obtained using binomial tests with Bonferroni corrections.

Multiple analyses of gene functions showed that the enriched gene functions did not overlap among the three clusters (Figure 4, upper panels). C1, C2, and C3 were highly enriched in genes related to transport (membrane), cell processes, and translation, respectively. This differentiation in gene function among the three clusters was commonly observed in all three annotation types. Additionally, the genes that were largely concentrated in C3 displayed structural component functions (mostly ribosome-related genes (Figure 4, green)) and were consistent with the essential nature of C3 genes (Figure 3A).

Additionally, no overlap was detected in the enriched regulations (TFs) between the three clusters (Figure 4, bottom panels). Both regulators and sigma factors were separately responsible for the three clusters. For instance, the genes regulated by *rpoN*, *rpoD,* and *rpoS* largely appeared in the C1, C2, and C3 clusters, respectively. This finding indicates that tasks are divided among the regulators that contribute to C1, C2, and C3. The regulator itself was not always clustered in the same cluster as the factors that it regulates (Table 1). For example, a considerable number of genes regulated by *rcsB* were clustered in C2, but *rcsB* itself was clustered in C3, supporting the negative transcriptional change relationship between C2 and C3 (Figure 3B).

**Table 1:** Gene clusters assigned to TFs and regulated genes

**TF name**	**Regulated cluster**	**TF cluster**
*rpoN*	C1	C2
*narP*	C1	C1
*narL*	C1	C3
*fhlA*	C1	C1
*rpoS*	C2	C2
*gadW*	C2	C2
*rcsB*	C2	C3
*gadX*	C2	C2
*rpoD*	C3	C1
*fis*	C3	C3
*purR*	C3	C3
*lrp*	C3	C3

TF name, Regulated cluster, and TF cluster represent the names of the regulators for the transcriptional networks (TFs), the clusters (defined in Figure 3) of the genes under the control of the corresponding regulators, and the clusters of the assigned regulators.

### Correlations between growth rates and changes in gene expression

**Figure 5:**
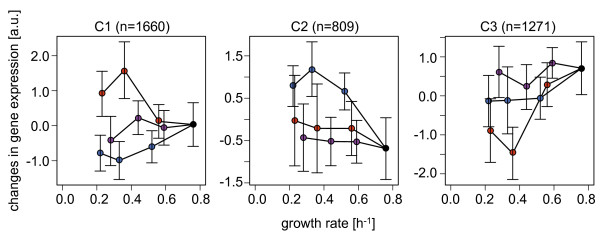
**Correlations between changes in gene expression and growth rates.** The three gene clusters (C1–3) determined by *K*-means clustering (*K* = 3) are shown in relation to growth rates. The mean values of the normalized transcriptional changes for all genes within the cluster are plotted against growth rates. The standard deviations represent the variation among genes within the same cluster. Black circles represent the expression levels measured under regular conditions. Differences in color saturation represent differences in growth rate. The use of color hue and saturation are as described in Figure 1: osmotic pressure (blue), temperature (red), and starvation (violet).

A correlation between gene expression changes and growth rates was evaluated on the basis of three gene clusters. The representative values for the average transcriptional changes (Figure 3B) were normalized as described in the Methods section, and the growth rates were determined as shown in Figure 1. Intriguingly, statistically significant correlations between the growth rate and changes in gene expression were clearly observed (Table 2), despite the fact that the clustering analysis did not account for growth. The transcriptional changes in genes from the C2 and C3 clusters exhibited negative and positive correlations with growth rate, respectively (Figure 5, middle and right). These correlations were commonly observed in all three types of environmental controls; that is, growth-related transcriptional changes were universal in the genes assigned to C2 and C3 and were independent of the types of environmental controls examined. On the other hand, the genes clustered in C1 exhibited distinct correlation directions with growth rates, which were positive in the osmo and strv conditions but negative in the heat conditions (Figure 5, left). Note that even when the number of genes was reduced from 3,740 to 2,805 following the removal of the low expression data sets, the *K*-means clustering analysis provided the same conclusion (Additional file 1: Figure S5). Additionally, the *K*-means analysis that clustered with a larger number of gene clusters (*K* = 16) toward the current data sets exhibited the same correlation trend (Additional file 1: Figure S6 and Additional file 1: Table S1).

**Table 2:** Correlations between growth rates and changes in gene expression

**Cluster**	***conc.***	***cor***	***p *** **value**
C1	osmo	0.56	0.00
C1	heat	-0.53	0.00
C1	strv	0.24	0.00
C2	osmo	-0.71	0.00
C2	heat	-0.22	5.24 × 10^-36^
C2	strv	-0.11	9.09 × 10^-10^
C3	osmo	0.41	0.00
C3	heat	0.73	0.00
C3	strv	0.17	0.00

The gene cluster (cluster) and the environment (*conc.*) represent the clusters divided as described in Figure 3 and the three types of environmental controls, respectively. The statistical significance of the correlations between the growth rates and the changes in gene expression are represented using the statistical significance (*p* value) and the correlation coefficient (*cor*) values.

The positive or negative correlations shared by clusters C2 and C3 indicate that the gene expression changes went in the same direction, independent of environmental diversity; this finding is consistent with the results shown in Figure 2. A common gene expression induction and repression occurred in C2 and C3 and was not biased by the initial expression under regular conditions (Additional file 1: Figure S7 and Additional file 1: Table S2). The difference in slopes for both positive and negative correlations within clusters C1, C2, and C3 implies an environmental specificity derived from osmotic pressure, temperature, and starvation. In addition, a principal component analysis (PCA) showed that PC1 and PC2 divided the expression patterns into three directions corresponding to the environmental types (Additional file 1: Figure S8, upper panels), which reflected the C1 cluster patterns (Figure 5, left). PC3 was correlated with growth rate (*cor* = -0.734, *p* = 0.016) with a contribution of approximately 5% (Additional file 1: Figure S8, lower panels). Therefore, the PCA analysis partially supported the conclusions drawn from the *K*-means clustering analysis but failed to illustrate a global confirmation of the correlation between growth and expression.

## Discussion

**Figure 6:**
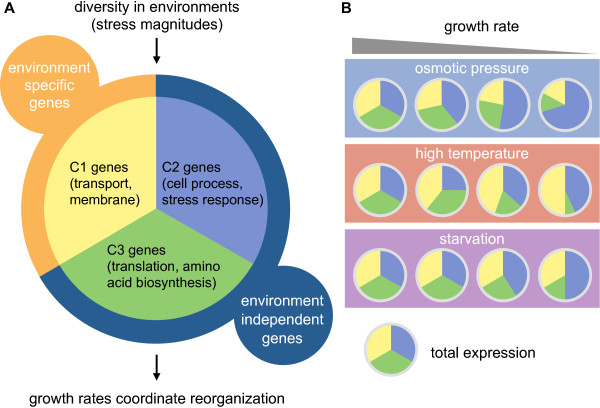
**Growth coordinated with transcriptome reorganization.** Exponentially growing cells comprise the three gene clusters **(A)** as follows: one cluster comprises environment-specific genes (orange), and two clusters comprise environment-nonspecific genes (navy). Clusters C1, C2, and C3 are noted in Figure 3. Balanced changes in gene expression among the three clusters are illustrated in coordination with growth rates **(B)**.

This study reports that 3,740 genes in an exponentially growing cell adjusted their expression levels in accordance with the cell division rate, either positively or negatively (Figure 5). Balanced transcriptional changes were observed among the gene clusters (Figure 6). The direction of transcriptional changes in C1 was environment-specific (Figure 6A); that is, the amount of C1 expression (yellow) was altered depending on the environmental type (Figure 6B). Changes in the C2 (blue) and C3 (green) expression levels ran counter to one another, regardless of environmental variation. Increased C2 expression was accompanied by decreased C3 expression and was coordinated with a reduction in growth rate (Figure 6B). The coherent dynamics of the C3 gene upregulation and the C2 downregulation indicated co-operation between gene clusters. No specialized mechanisms have been reported in bacterial cells to explain this phenomenon as yet, although the specific pathways initiated by SAPK and TOR are known in eukaryotic cells [9,10]. The new finding of a balanced relationship in bacterial gene expression indicates the existence of an energy saving and/or resource conservation strategy in transcriptome reorganization, which may help to maintain the homeostatic framework in cellular physiology, as predicted by in silico biochemical networks [38].

This balanced lifestyle (Figure 6) was largely attributed to differentiation in gene function among the three gene clusters (Figure 4). The C1 genes were largely enriched in membrane proteins and transporters, suggesting that these genes are the first responsive sensors to all types of environments (Figure 6A). The C2 genes shared a common decrease with increased growth (Figure 5) and a general stress response function (Figure 4), which plays a central role in cellular processes. The difference in negative correlation slopes may indicate a diversity of molecular mechanisms under the three environmental types; osmotic pressure might be regulated by two-component systems [39], growth at high temperatures might induce heat shock proteins [26], and growth under starved conditions might activate the stringent response [30,40]. The genes clustered in C3 were positive correlated with growth rate (Figure 5) and were largely related to ribosome translation machineries (Figure 4). A correlation between the growth rate and the quantity of ribosomes was previously reported in cells growing under starvation conditions [41,42]. A global view of the coordination of growth rate with transcriptional changes as presented here illustrates a vivid picture of the differentiated gene expression balance in sustaining life under diverse habitats.

Here, we focused on constant growth states (on a time scale of approximately 10 hours of exponential growth) rather than the stress response phase (Additional file 1: Figure S1). Despite the divergence in time scales between the two phases, both types of transcriptome reorganization presented relationships between transcriptional fluctuations and the degree or magnitude of stress. Some common results were found, such as similar changes in transcriptional networks (Figure 2 and Additional file 1: Figure S4). A few highly sensitive TFs were found, including *purR* and *rcsB* (Figure 2 and Additional file 1: Figure S4). Because these TFs were also found to be sensitive to genome reduction [15], it appeared that these TFs responded to all types of perturbations, both internal and external, and were highly sensitive to phases of stress response and exponential growth.

However, the direction of transcriptional changes in the three gene clusters exhibited weak similarity in terms of stress response and exponential growth. Using the reported data sets on the stress response [24], we found that only C3 genes experienced a common decrease in expression under all four stress conditions (Additional file 1: Figure S9); this finding is consistent with the positive correlation of C3 with growth rates (Figure 5). Furthermore, applying the same *K*-means clustering did not yield a clear correlation between growth rates and changes in gene expression during periods of stress response (Additional file 1: Figure S10), which is consistent with findings in yeast [25]. Taken together, survival strategies in bacteria faced with pulse-like perturbations and continuous growth in new environments were partially shared, but the coordination with growth rate was apparent only in exponentially growing cells and was not present in responsive cells during stress response.

## Conclusions

Here, we provided the first experimental evidence for coordination between constant growth rates and changes in gene expression, which is a general phenomenon that applies to many environments. Growth rate-associated gene function differentiation and gene regulation task division were defined. This experiment was successful in categorizing 3,740 genes across the genome into three clusters and in matching the changes in gene expression patterns with the growth rate. This success resulted from using an experimental design that primarily targeted the exponential phase and from the use of a clean genome without any insertion sequences [43] that could have masked transcriptional change patterns.

## Methods

### Strain, media and culture conditions

The genetically engineered *E. coli* strain *MDS42* Δ*galk::P*_*tet*_*-gfp-kan* was used. *Gfp* and *kan* marker genes were introduced into the genome-reduced strain MDS42 [43]. The *gfp* reporter gene was used to facilitate cell counting by flow cytometry, and the kanamycin-resistance gene *kan* was used as a selection marker. These marker genes were inserted at the *galK* site by homologous recombination as previously described [44,45]. The cells were aerobically cultured with 5 ml of M63 minimal medium (62 mM K_2_HPO_4_, 39 mM KH_2_PO_4_, 15 mM (NH_4_)_2_SO_4_, 2 μM FeSO_4_ · 7H_2_O, 15 μM thiamine hydrochloride, 203 μM MgSO_4_ · 7H_2_O, and 22 mM glucose) at 37°C in a shaking water bath (Personal 11, Taitec) at 160 rpm. Cells were inoculated into fresh medium from a glycerol stock and then pre-cultured overnight. The exponentially growing cells were subsequently transferred to a series of environments; that is, into ten different culture conditions. One set of conditions (the same as those used in the pre-culture described above) was used as a control. Three different osmotic pressure conditions were used: 0.2 (osmo 1), 0.45 (osmo 2), and 0.55 M (osmo 3) of NaCl (Wako) in minimal media. Three different growth temperatures were used (in minimal media): 40.0 (heat 1), 41.5 (heat 2), and 41.8°C (heat 3). Finally, three serine-depleted media were used: 50 μg/ml (strv 1), 100 μg/ml (strv 2), and 150 μg/ml (strv 3) of DL-serine hydroxamate (SHX; Sigma) in minimal media.

### Cell culture and growth rate

*E. coli* cells were measured using a flow cytometer (FACSCanto II; Becton-Dickinson) equipped with a 488-nm argon laser and a 515–545-nm emission filter (GFP). The following PMT voltage settings were applied: forward scatter (FSC), 300; side scatter (SSC), 400; and GFP, 600. The flow rate for the sample measurements was set to ‘low’. Cell concentrations were calculated according to the ratio of gated particles representing the number of *E. coli* cells carrying the reporter gene *gfp* and beads of known concentrations, as previously described [45]. Temporal changes in the cell concentration under the ten studied culture conditions were monitored at a time interval of approximately 2 hours (data not shown). Based on the growth curves obtained, the growth rates of the cells within the early exponential growth phase were determined (Additional file 1: Figure S2). The growth rates were calculated according to the initial and final cell concentrations and the culturing time, as previously described [15]. According to growth rates measured in preliminary experiments (Additional file 1: Figure S2), independent cell cultures were applied repeatedly for all 10 conditions, and cells within the early exponential growth phase were collected for further microarray analysis. The initial cell densities were 1–9 × 10^6^ cells/ml, and the final cell densities were maintained at approximately 2 × 10^8^ cells/ml. The exponential growth time lasted 6–10 hours.

### Microarrays and expression data normalization

Three independent cell cultures were used for the microarray analysis to acquire the mean expression under each culture condition. The preparation of total RNA samples and microarray analysis using an Affymetrix GeneChip system were performed as described elsewhere [46]. A high-density DNA microarray covering the entire *E. coli* W3110 strain genome (GenoBase; http://ecoli.naist.jp/GB6/search.jsp) was used as described [15,46]. The number of genes and the strain gene names used here were selected based on a previous report [15]. The finite hybridization model was applied to extracted raw data on a logarithmic scale, as previously described [47,48]. Three biological replicates were obtained under each condition described above. Gene duplicates were unified according to the gene name annotation as the mean values of the duplicate expression levels. Expression data sets from biological replicates are shown as the mean values. The raw data sets were subjected to global normalization, resulting in a common median value of zero (logarithmic value) in all data sets. Both the normalized expression data sets and the raw CEL files were deposited in the NCBI Gene Expression Omnibus database under the GEO Series accession number GSE49296 (http://www.ncbi.nlm.nih.gov/geo/query/acc.cgi?acc=GSE49296).

### Computational analysis

Binomial tests were performed to evaluate the statistical significance of the extracted gene groups. These statistical analyses were carried out using free software packages available from the Broad Institute (http://www.broadinstitute.org). All statistical tests and computational analyses except for the gene sets enrichment analysis (GSEA) were performed using R [49]. GSEA [31] was performed as previously described [15]. *K*-means clustering [34] was performed for all 3,740 genes, and the expression levels acquired under 10 culture conditions were normalized to a common mean value of zero and a variance of one. The gene expression level (logarithmic scale) obtained under the regular condition was subtracted from the three types of environmental controls (osmo 1–3, heat 1–3 and strv 1–3). Consequently, nine transcriptional change values and a base value of zero (regular condition) were acquired for each gene. *K*-means clustering analysis was performed on this data set comprising 10 × 3,740 values. PCA was performed according to previous reports [50,51], which classified expression patterns according to gene expression level variance between the culturing conditions.

### Annotations of gene function and regulation

Transcriptional network information for *E. coli*, including regulators (*i.e.*, sigma factors and transcriptional factors) and downstream regulated genes, were obtained from RegulonDB 8.0 [33] (http://regulondb.ccg.unam.mx). Forty transcriptional networks (comprising more than 15 regulated genes controlled by a regulator) were used in the analysis. MultiFun annotation was carried out according to the GenProtEC database [37] (http://genprotec.mbl.edu). The MultiFun classification was applied according to the top two hierarchical levels. These gene categories have been discussed in a previous report [32]. Twenty-one gene categories (comprising more than 10 genes within each category) were used in the analysis. A GO term annotation of *E. coli* strain K-12 was obtained from the Gene Ontology database [35,36] (http://www.geneontology.org). GO terms that comprised less than 15 genes or more than 1,000 genes were neglected in the analysis. Gene synonyms were entered into EcoCyc [52] (http://www.biocyc.org), and the resulting synonyms were integrated into a single term by averaging their expression levels. All annotations were reciprocally associated and comprehensively re-mapped.

### Availability of supporting data section

http://www.ncbi.nlm.nih.gov/geo/query/acc.cgi?acc=GSE49296.

## Competing interests

The authors declare that they have no competing interests.

## Authors’ contributions

BWY and TY conceived the research. YMa performed experiments. YMa and BWY analyzed the data. BWY and YMa wrote the paper. YMu, ST, BWY and TY provided experimental and analytical tools. All authors read and approved the final manuscript.

## Supplementary Material

Additional file 1 **Supplementary Information. Figures S1–S10** and **Tables S1–S2**. Click here for file
