# Supplementary material for: Growth rate-coordinated transcriptome reorganization in bacteria
Source: BMC Genomics. 2013 Nov 20;14:808. doi: 10.1186/1471-2164-14-808 (PMC3840594; doi:10.1186/1471-2164-14-808)
Supplement: Additional file 1 — Supplementary Information. Figures S1–S10 and Tables S1–S2. [file 1471-2164-14-808-S1.pdf]

Growth rate-coordinated transcriptome reorganization in bacteria

Yuki Matsumoto *et al.*

Supporting Information

I. Supplementary Figures and Figure Legends (Figures S1–S10) **p. 2-11**

II. Supplementary Tables (Tables S1–S2) **p. 12-13**

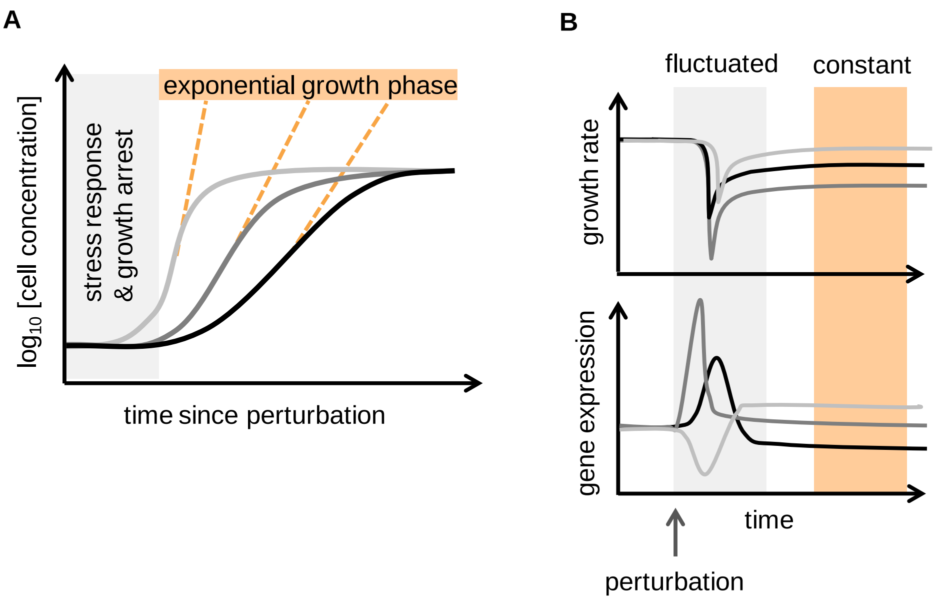

Figure S1. Stress response, gene expression, and cell growth. A. Growth curves under varied environments. Two distinct periods are indicated: stress response (gray) and constant growth (orange). Pulse-like transcriptional changes in response to perturbations generally run from several minutes to a few hours in phases designated as stress response. The later exponentially growing phase is designated as the period of constant growth. **B.** Fluctuating and constant periods of growth and expression. A certain stress response induced by a certain environmental change not only causes changes in gene expression but also arrests cell growth. The slope of the growth curves in the exponential phase (A) represents the growth rate (B). Three curves graded from black to light grey represent the changes in growth rates (upper panel) and transcriptional changes of an identical gene (bottom panel) under three different environments. The fluctuating (grey shadow) and constant (orange shadow) periods correspond to the stress response period and the exponential growth phase in A, respectively. The arrow indicates the moment at which the environmental changes occurred.

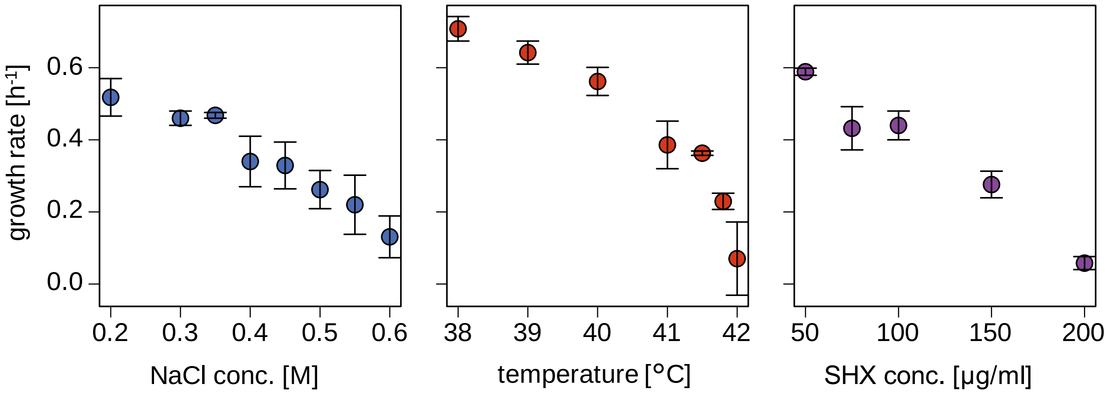

Figure S2. Growth rates under various conditions. The growth rates of exponentially growing cells in the three types of environments studied are shown. Increased concentrations of NaCl (blue) or DL-serine hydroxamate (SHX, violet) result in raised osmotic pressure or enhanced starvation, respectively, leading to decreased growth. Increases in temperature (red) decrease growth rates. The quantitative relationships between growth rate and culturing conditions are shown. The experimental errors obtained based on the repeated cultures are indicated.

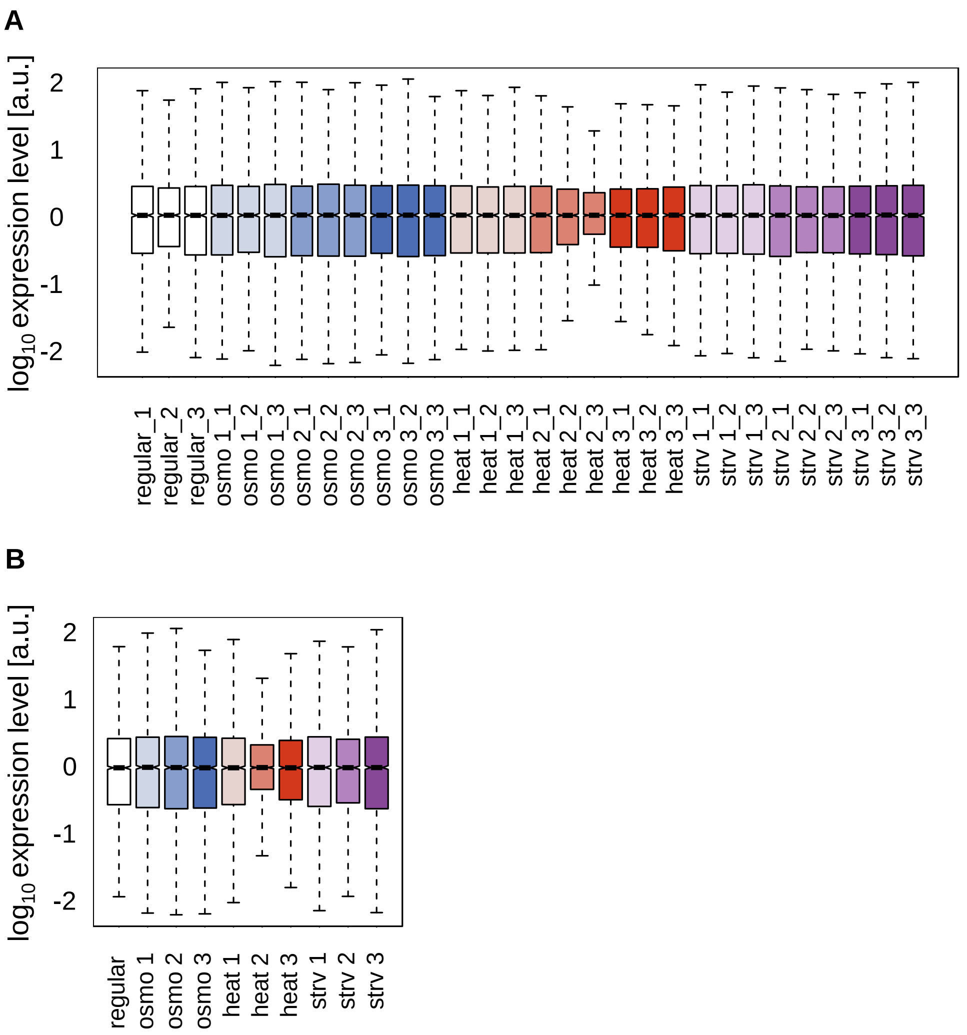

Figure S3. Box plots of gene expression. Individual results from repeated experiments (in triplicate for each condition (A) and averaged expression data sets (B) are shown. The expression of 3,740 genes under regular (regular), osmotic pressure (osmo), raised temperature (heat) or starved (strv) conditions are indicated. The white box represents the expression measured under regular conditions (regular). The color variation is corresponds to the growth conditions as in Figure 1. The differences in color saturation represent differences in growth rate. The expression levels represent log-scale mRNA concentrations (a.u.).

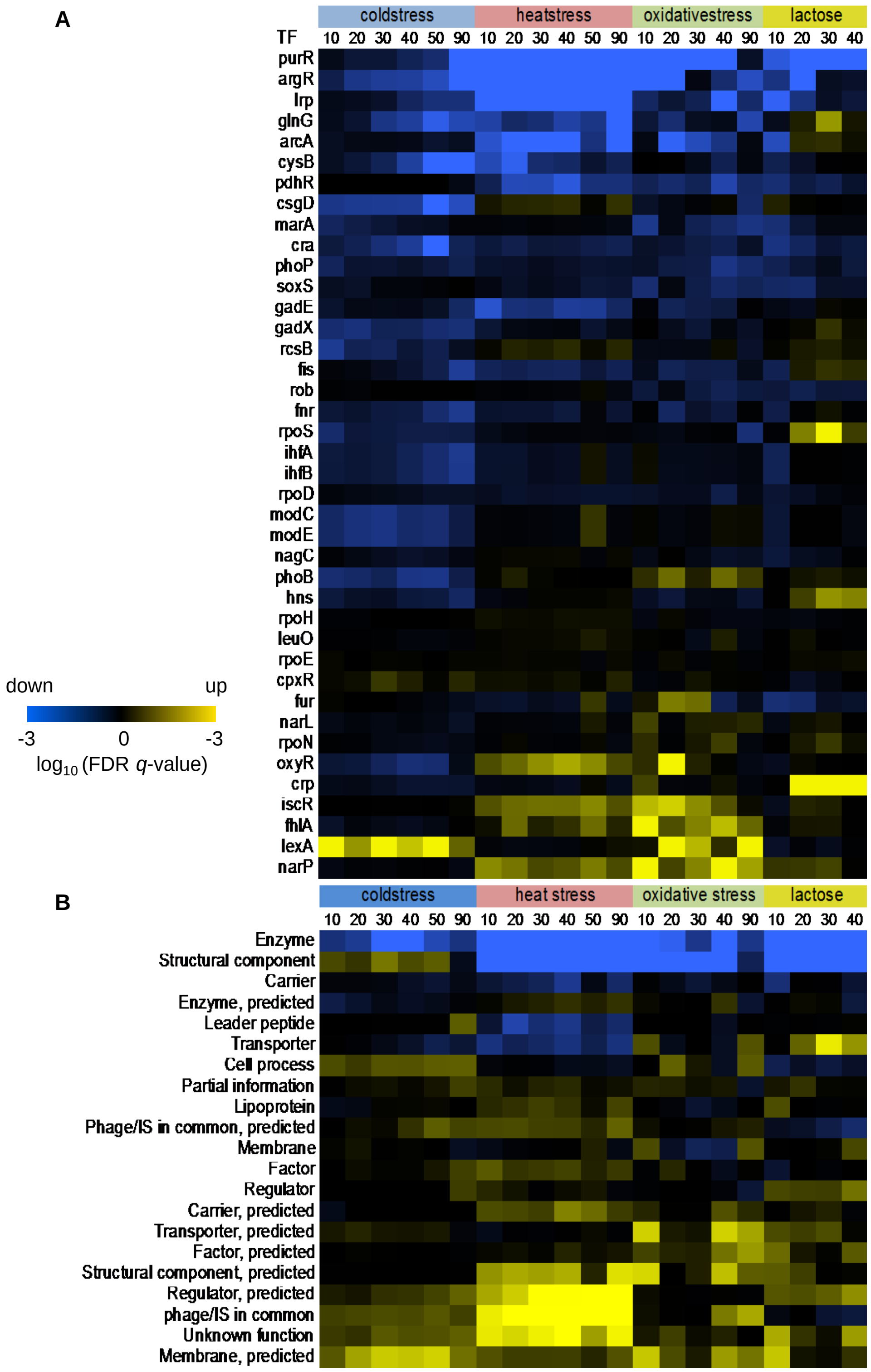

Figure S4. Transcriptional changes in stress response. GSEA was performed using the data sets of responses to cold stress, heat stress, oxidative stress and lactose [24]. Two types of annotations (TFs (A) and gene categories (B)) were carried out to enrich the gene regulation and function. Four types of stresses are indicated using different colors, and the time sampling of the *E. coli* cells suffering from stress are noted using numbers. The statistical significance (FDR *q*-value) of the transcriptional changes in TFs and gene categories is represented on a logarithmic scale using a color gradation from dark brown to yellow or blue. Vivid colors represent high significance in the direction of either upregulated (yellow) or downregulated (blue) genes.

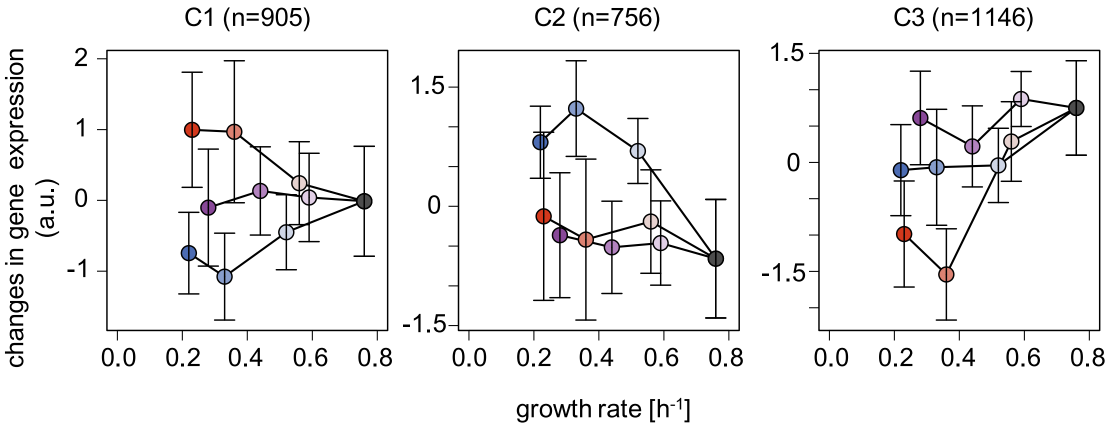

Figure S5. *K*-means clustering analysis using a reduced number of genes. Poorly expressed genes (p<0.5) were removed from the analysis, and the expression data sets for 2,805 genes were used. The analytical procedure used was the same as that described in Figure 5. The number of genes classified into three gene clusters (C1–3) is indicated. The standard deviations and color hue and saturation are as indicated in Figure 5.

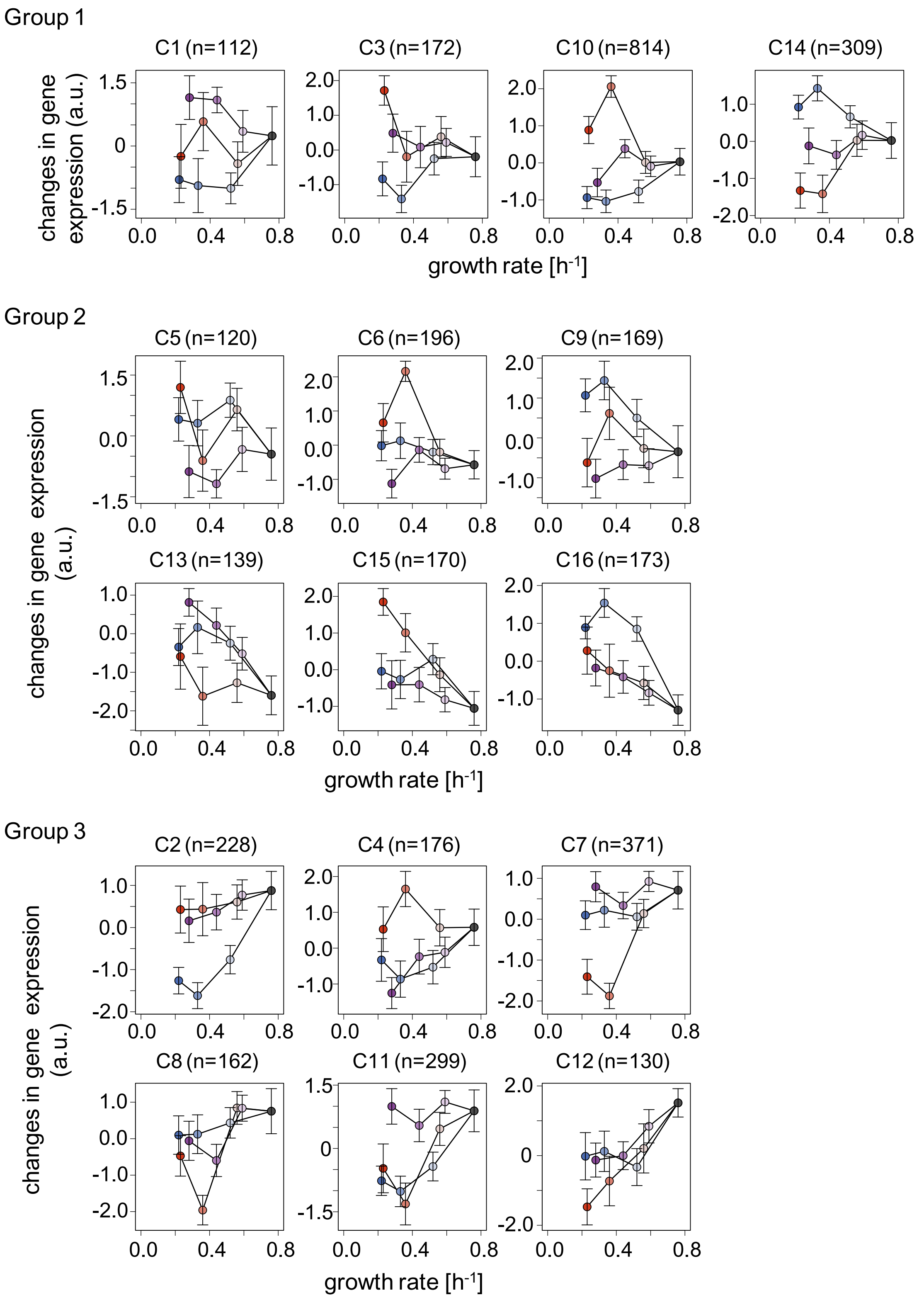

Figure S6. *K*-means clustering analysis. The analytical results of K=16 are shown. Sixteen gene clusters (C1–16) were defined, comprising various numbers of genes (indicated). These clusters were further categorized into three groups (Groups 1–3) according to the correlation coefficients, as summarized in Table S1. The standard deviations and color hue and saturation are as indicated in Figure 5. Both positive and negative correlations between the growth rates and the changes in gene expression were found in all clusters, and further categorization resulted in different correlations among the three groups; positive in Group 3, negative in Group 2, and environment-specific in Group 1. This trend was consistent with the results of K=3, as shown in Figure 5.

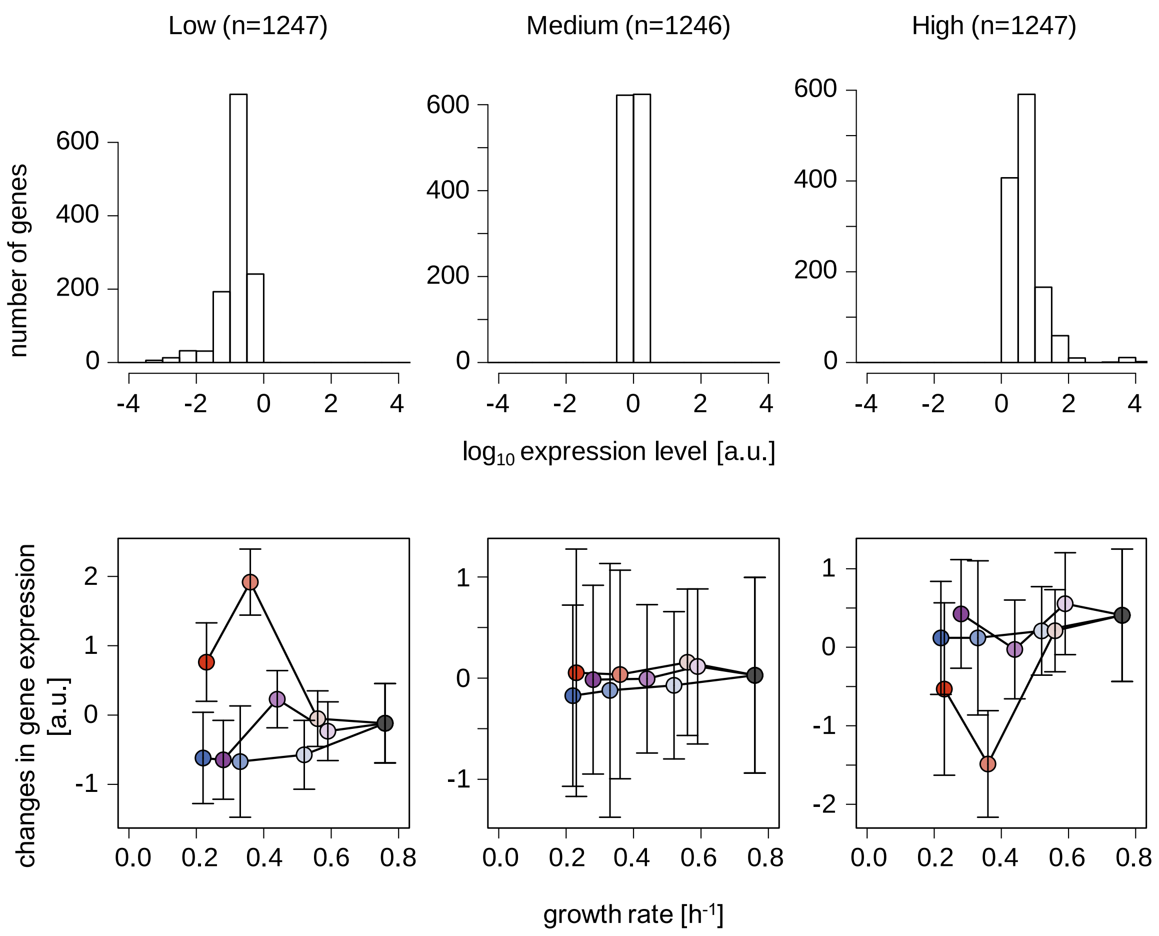

Figure S7. Gene categorization according to regular expression. A total of 3,740 genes were divided into three clusters, each containing approximately the same number of genes (as indicated), according to their expression level under regular conditions. The distributions in expression level for the gene clusters with low, medium and high expression levels are shown in (A). The averaged expression changes of the three gene clusters under the nine culture conditions are plotted against growth rate (B). The standard deviations and color hue and saturation are as indicated in Figure 5. With the exception of the expression under environments at different temperatures, no significant correlation to the growth rates was found in any of the three clusters (Table S2). These results suggested that the common trend of increased and decreased expression in C2 and C3 was not determined by the regular expression level.

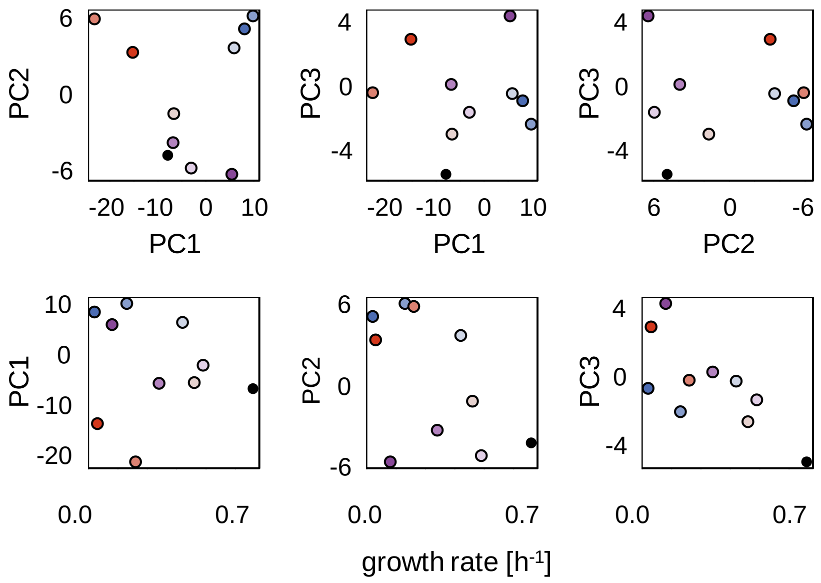

Figure S8. Principal component analysis (PCA). This analysis was performed based on gene expression levels. The order of PCs was determined by the variance in expression levels for all genes. Because the first three PCs exhibited a total variance of 0.910, representing more than 90% of the entire data set, only the results for PC1–3 are shown. The upper and lower panels show the relationships between pairs of PCs and between each PC and growth rate, respectively. The correlation coefficients, *p* values, and total variance of the three PCs are as follows: -0.138, 0.704, 0.700 (PC1); -0.521, 0.123, 0.862 (PC2); -0.734, 0.016, 0.910 (PC3).

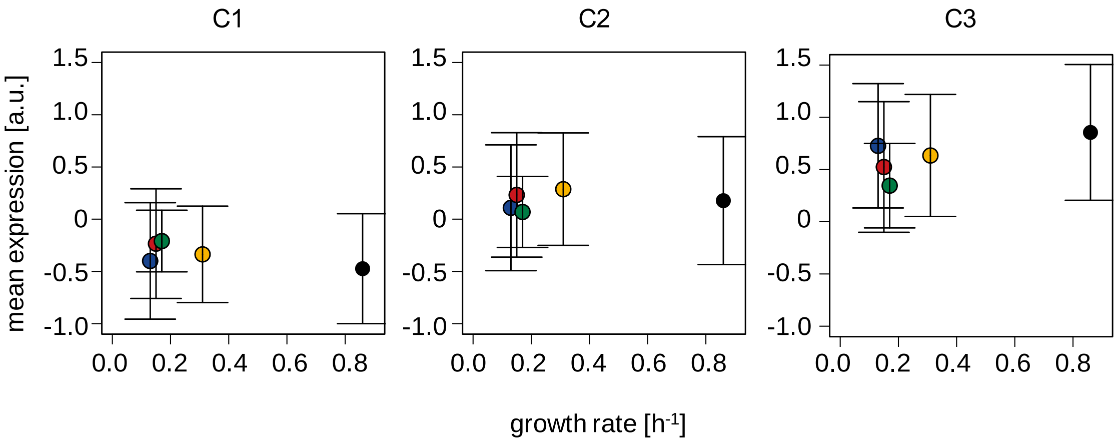

Figure S9. The relationship between transcriptional changes and growth rate during the stress response phase. Expression data sets recorded at 40 min (Fig. S4) were used. The growth rates were estimated based on the original paper [24]. Because the culture times were as short as 10–210 minutes, the estimated growth rates might be far from correct. The genes were assigned to the three clusters (C1–3) that were determined as shown in Figure 3. The mean expression levels of genes within the same clusters are plotted against estimated growth rates. Red, blue, green, and orange represent the averaged gene expressions under heat stress, cold stress, oxidative stress, and lactose. The standard deviations in expression levels among genes within the same clusters are shown.

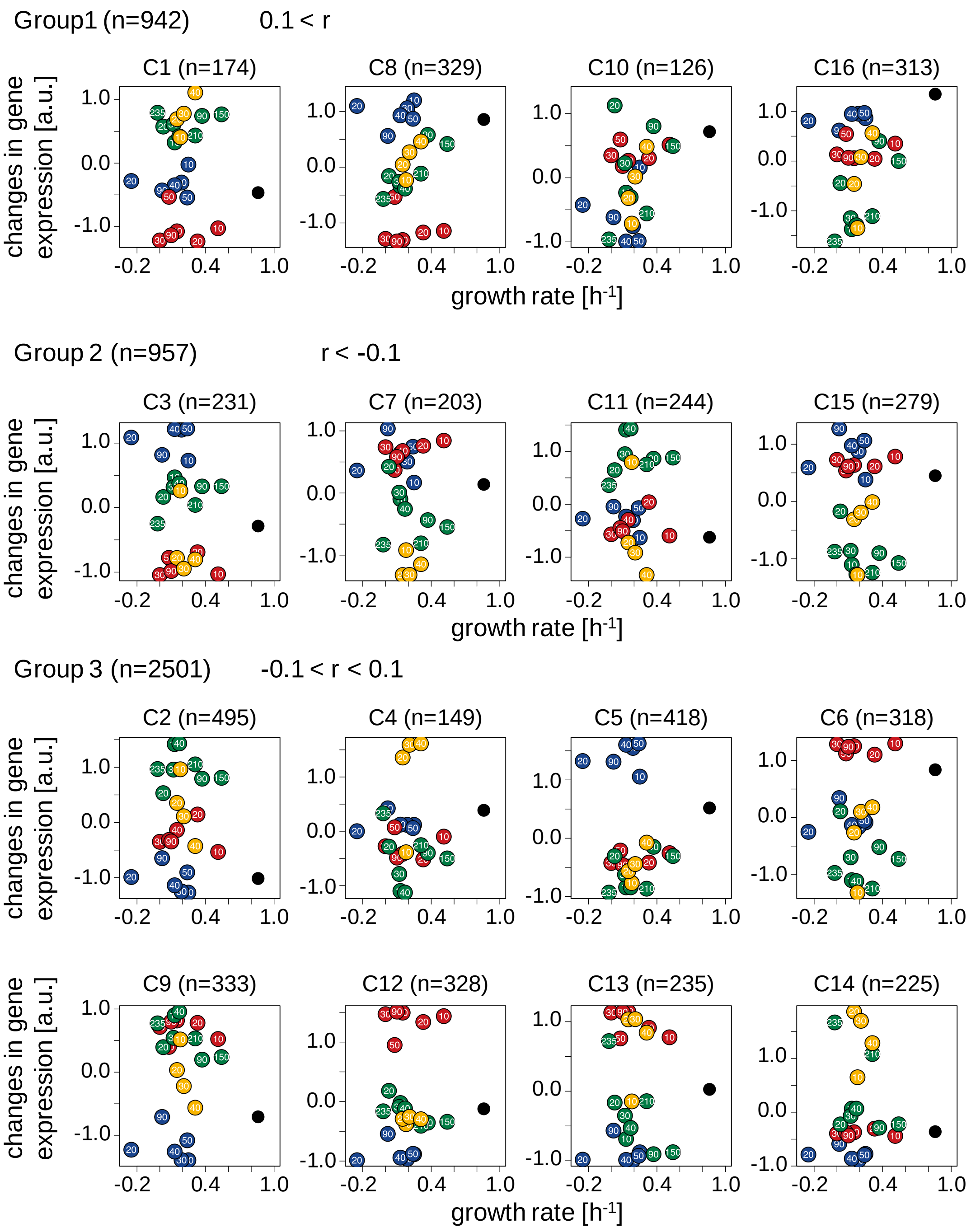

Figure S10. *K*-means clustering to the data sets with respect to stress response. The data sets in Figure S4 were applied to the *K*-means clustering analysis as described in Figure S6 (*K*=16) according to changes in gene expression between the stress response experiments and the control. Sixteen gene clusters (C1–16) were defined, comprising various numbers of genes (indicated). These clusters were further categorized into three groups (Groups 1–3) according to the correlation coefficients as indicated. The growth rates and color variations are described in Figure S9. The numbers indicated in the colored cycles represent the time sampling points according to the original report [24]. No significant correlations between changes in gene expression and growth rates were detected.

Table S1. Categorization of gene clusters. Sixteen gene clusters defined by *K*-means clustering analysis (as shown in Fig. S6) were categorized into three groups (Groups 1, 2, and 3) according to the correlation coefficients between the growth rates and the changes in gene expression (*cor*). The number of gene clusters (Cluster), the correlation coefficients (*cor*), and the respective *p* values are shown.

Group	Cluster	*cor*	*p* value
1	1	0.06	3.8×10^{-2}
(-0.1 < *cor* < 0.1)	3	-0.06	2.2×10^{-2}
	10	-0.01	5.7×10^{-1}
	14	0.09	1.0×10^{-6}
2	5	-0.15	4.1×10^{-7}
(*cor* < -0.1)	6	-0.29	1.4×10^{-40}
	9	-0.22	1.4×10^{-20}
	13	-0.40	2.7×10^{-54}
	15	-0.51	1.8×10^{-115}
	16	-0.57	1.1×10^{-152}
3	2	0.45	0.0
(*cor* > 0.1)	4	0.20	0.0
	7	0.41	0.0
	8	0.46	0.0
	11	0.50	0.0
	12	0.62	0.0

Table S2. Correlations between growth rates and changes in gene expression. The gene cluster (cluster) was divided according to the regular expression level (as shown in Fig. S7). L, M, and H represent low, medium, and high expression levels under regular conditions, respectively. Environment (*conc.*), the statistical significance (*p* value), and the correlation coefficients (*cor*) are as described in Table 2.

**Cluster**	***conc.***	***cor***	***p* value**
L	osmo	0.29	0.00
L	heat	-0.58	0.00
L	strv	0.21	0.00
M	osmo	0.08	1.02×10^{-7}
M	heat	0.00	0.76
M	strv	0.03	2.63×10^{-2}
H	osmo	0.14	0.00
H	heat	0.50	0.00
H	strv	0.08	6.68×10^{-8}
